# Microfluidic Platform with Serpentine Geometry Providing Chaotic Mixing in Induction Time Experiments

**DOI:** 10.1021/acs.cgd.1c01436

**Published:** 2022-06-09

**Authors:** Sameer D. Shingte, Olav Altenburg, Peter J. T. Verheijen, Herman J. M. Kramer, Huseyin Burak Eral

**Affiliations:** † Process & Energy Department, Delft University of Technology, Leeghwaterstraat 39, 2628 CA Delft, The Netherlands; ‡ Biotechnology Department, Delft University of Technology, 2629 HZ Delft, The Netherlands; § Van’t Hoff Laboratory for Physical and Colloid Chemistry, Debye Institute, Utrecht University, Padualaan 8, 3584 CH Utrecht, The Netherlands

## Abstract

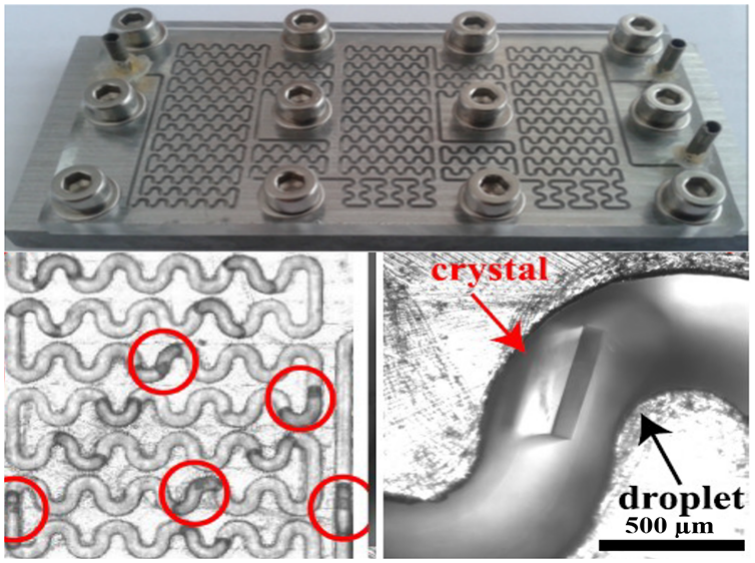

We present a droplet microfluidic platform mixing the contents of the droplet chaotically in microfluidic induction time measurements, a promising method for quantifying nucleation kinetics with minute amounts of solute. The nucleation kinetics of aqueous potassium chloride droplets dispersed in mineral oil without surfactants is quantified in the presence and absence of chaotic mixing. We demonstrate the ability of the proposed platform to dictate droplet size, to provide a homogeneous temperature distribution, and to chaotically mix the droplet contents. Chaotic mixing in induction time measurements is facilitated by the motion of droplets through serpentine micromixer bends, while the extent of mixing is controlled by how much droplets move. Different nucleation kinetics are observed in experiments where the droplets are static, mixed, and in motion. We hypothesize that the droplet motion induces formation of a thin-liquid Bretherton film surrounding the droplets. The thin film shields droplets from solid boundaries that are more efficient heteronucleant surfaces compared to liquid–liquid interfaces. We observed that repeated microfluidic induction time measurements, particularly with moving droplets, produce significantly distinct cumulative nucleation probability curves, indicating that the measured nucleation kinetics depend strongly on the details of the experimental procedure, which we discuss in detail. Finally, we compare the microfluidic experiments to well-mixed, milliliter volume, turbidity-based measurements in the context of classic nucleation theory.

## Abstract 2

We present a microfluidic device to examine the influence of chaotic mixing, caused by serpentine microchannels, on nucleation time in microfluidic droplets. This can be an effective tool for quantifying nucleation kinetics by consuming minute amounts of solute. Distinct nucleation kinetics observed with the static, mixed, and in-motion droplets is hypothesized to be influenced by a thin-liquid Bretherton film surrounding the droplets.

## Introduction

Crystallization from solution is a widely used separation and purification method in the production of crystalline solids ranging from pharmaceuticals to specialty chemicals.^1,2^ Our ability to accurately quantify nucleation kinetics plays a critical role in the design and control of industrial crystallization processes while offering mechanical insights into nucleation pathways. Nucleation is commonly quantified through induction time experiments^3^ where a large number of identical experiments are performed to account for the stochastic nature of nucleation.^4^ A large number of identical experiments ensure the statistical significance of experimental deductions.^5,6^ Various methods have been developed to measure induction times, including pendant drop, double pulse technique, levitated drop, camera, and turbidity-based methods.^1,3,7−9^

Among these methods, measuring nucleation rates in droplets has emerged as a promising method.^3,10−12^ The idea of using small droplets for induction time measurements was presented already in 1959 by White and Frost.^13^ White and Frost dripped small droplets of an aqueous solution in mineral oil and performed induction time measurements. These droplets had diameters varying between 0.2 and 2 mm. With the advent of micromanufacturing, droplet microfluidics emerged as a cost-effective method for creating a large number of droplets with controlled size distribution while using minute amounts of solute—an important feature while working with difficult to synthesize or expensive compounds. By balancing capillary forces dominant on the micrometer scale with viscous forces,^14,15^ droplet-based microfluidic platforms can provide a large number of droplets (100–1000 per device) acting as identical isolated crystallization reactors to ensure statistical accuracy.^10,11^ Particularly, droplet volume, a critical parameter in classic nucleation theory,^4^ can be tightly controlled in droplet microfluidics.^16^ Although other approaches have been proposed,^3,17−20^ in a typical microfluidic induction time experiment, droplets carrying known concentrations of solute are produced and then cooled down to reach a given supersaturation. At this prescribed supersaturation, the nucleation kinetics are quantified by two different approaches with significantly different mixing behaviors. In the first approach, the static droplets are stored at a fixed supersaturation, and all the droplets are observed until the end of the experiment to quantify the fraction of crystallized droplets as a function of time.^21−23^ This fraction is used to construct the cumulative nucleation probability function, *P*(*t*), correlated to the induction time and nucleation rate.^6,24^ In the second approach, moving droplets in a capillary or microfluidic channel are observed at a given observation time, *t*_obs_, after a prescribed supersaturation is reached. The fraction of crystal-containing droplets at this fixed *t*_obs_ is quantified optically as droplets flow. Varying this fixed *t*_obs_ allows construction of the cumulative nucleation probability function and quantification of nucleation kinetics; yet, these measurements are often reported at a single *t*_obs_.^16,25^ In both approaches, emergence of the first crystal is detected by video microscopy. The fundamental difference between these two approaches is that the droplets are static in the first approach,^21−23^ whereas they are moved but not actively mixed in the second one.^16,25^ These two microfluidic approaches rarely produce the same nucleation rates even for identical solutes under identical conditions. Moreover, microfluidic nucleation rates rarely match turbidity-based measurements. We hypothesize that hydrodynamics and surface interactions may play a role in explaining this discrepancy. In one of the earlier studies, Zheng et al.^21^ demonstrated crystallization in the stagnant droplets at a constant temperature for protein screening applications. Following this study, microfluidic induction time measurements of droplets containing various solutes^22,23^ and the nature of the oil–water interface have also been investigated.^26^ Ildefonso et al.^26,27^ studied heterogeneous nucleation and the emergence of polymorphs in stagnant microfluidic droplets. Grossier et al.^28^ developed an approach where nucleation is induced with a sharp tip in micron-scale droplets through contact or electric fields. More recently, Dos Santos et al.^16^ characterized nucleation and growth rates in moving droplets by varying the supersaturation and residence time while accounting for the droplet volume distribution and the uncertainty in the automated image analysis procedure.

The influence of hydrodynamics in microfluidic induction time measurements has attracted attention recently. Rossi et al.^29^ studied the effect of flow comparing the nucleation in stagnant and flowing droplets where the contents of droplets are mixed by symmetric advection rolls. The authors showed a significant increase in nucleation rates in droplets that were moving compared to static droplets. The authors concluded that the internal circulation caused by the movement of the droplets affects the kinetic parameter of the nucleation expression without affecting the thermodynamic parameters. Nappo et al.^30^ investigated the effect of shear rate on the nucleation by comparing induction times in stagnant and moving droplets in a microfluidic device with those in stirred vials of 2 mL. These authors observed a very strong increase of the nucleation rate in moving compared to static droplets, while somewhat lower nucleation rates were found in the stirred vials, despite a two orders of magnitude higher shear rate in the stirred vials. On a separate note, Dela Cruz et al.^31^ concluded that changes in the shape of the probability distribution is an indication of the change in the nucleation regimes, which in most cases is caused by polymorphism, i.e., because of concomitant nucleation of two polymorphic forms or by transformation of a metastable into a more stable polymorph. A more extensive review of microfluidic induction time measurements can be found in following review articles.^3,10,11^

As the nucleation rate is reported per unit volume, one may be misled to think nucleation rates measured in microfluidics may be directly implemented in industrial-scale crystallizers. It is important to emphasize that industrial crystallization processes take place in larger volumes not only but also in fundamentally different hydrodynamic environments.^1^ Hydrodynamics inside industrial crystallizers affects the growth rates as well as other hydrodynamics-induced phenomena such as secondary nucleation, attrition, and aggregation rates.^2,32,33^ Hence, the “effective” nucleation rates obtained from microfluidics that include contributions from both nucleation and highly hydrodynamics dependent growth cannot be used for designing and controlling industrial-scale models. Nonetheless, development of a microfluidic platform with precisely controlled mixing can shed light on the often overlooked role that these two phenomena play in microfluidic induction time measurements.

Although microfluidics shows a great deal of potential for various applications, it also suffers from a number of drawbacks, e.g., limited mixing and the dispersion of constituents.^34^ Microfluidic flows are laminar; hence, the absence of turbulence results in poor component mixing in the microchannel. Turbulence can be induced by increasing the fluid flow rate. This, however, may lead to excessive consumption of the chemical components, which undermines one of the key advantages of microfluidics. The other way of inducing rigorous mixing is introducing chaotic advection through the geometry of the channel. There are different strategies to induce chaotic advection: one of them is to design a serpentine geometry for the microchannel, as reported by Bringer et al.^34^ and Harshe et al.^35^ The unique geometry of the microchannel enhances mixing by successive cycles of stretching, folding, and reorientation of substance molecules in the fluid.^14^ These successive cycles resemble the baker’s transformation during dough formation. Therefore, mixing is facilitated as droplets move along the channel. When the droplet moves in the straight microchannel, symmetric eddies are generated, but there is no effective mass transfer taking place between the two halves of the droplet, which leads to limited mixing. Conversely, asymmetric recirculation flows are developed inside the droplet moving through the winding microchannel. They give rise to chaotic advection leading to chaotic mixing. Therefore, enhanced mixing within the droplets is expected using the device presented in this work compared to the microfluidic systems reported by Nappo et al.^30^ and Rossi et al.^29^

The purpose of this work is to present a microfluidic platform for induction time measurements where the contents of the dispersed droplets are mixed by chaotic advection initiated by the serpentine shape of the device. We share our experiences including experimental difficulties and challenges in interpreting observations in a candid and straightforward manner. The proposed platform offers the possibility to manipulate the hydrodynamics and mixing conditions inside the droplets through droplet motion. Consequently, the platform enables studying the intertwined role of droplet motion and mixing on nucleation kinetics in microfluidic experiments. Moreover, the device builds upon the well-documented advantages of microfluidic design, such as ability to perform high-throughput experiments with minute amounts of material screening at low material costs (low volume) with excellent control of process conditions due to high mass and heat transfer rates.

**Figure 1:**
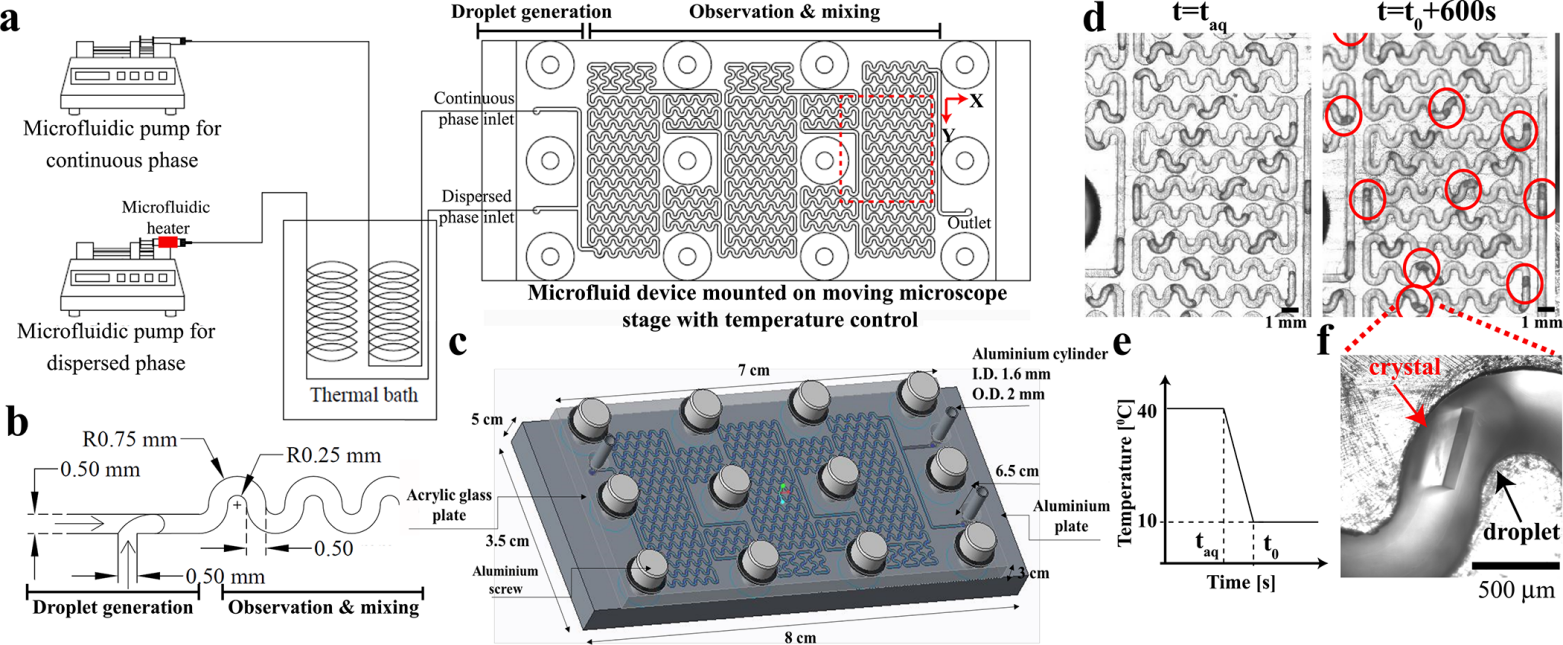
Experimental setup: (a) two syringe pumps, a syringe pump heater for the dispersed phase, a thermal bath, and the microfluidic device mounted on an inverted microscope, which visualized the droplets (red dashed square). The field of view is systematically translated in *X* and *Y* axes to image the whole device with an automated moving stage enabling the observation of ∼300 droplets in one experiment. (b) Details of the microchannel (0.5 mm wide and 1 mm or 0.5 mm deep) having a serpentine structure to enhance mixing with the illustration of droplet formation using a T-junction. (c) 3D view of the microfluidic device. (d) Bright field microscopy images of the field of view before and after reaching the target crystallization temperature. Droplets with crystals are the red circles. (e) Temperature profile. *t*_aq_ is the video acquisition time, and *t*_0_ is the moment reaching the target temperature. (f) An optical microscopy image of a droplet with a crystal inside.

The presented microfluidic platform (Figure 1) produces droplets carrying prescribed concentrations of a model solute, potassium chloride, KCl, without the need for surfactants and stores them for observation at an elevated temperature as an undersaturated solution. The stored droplets are rapidly cooled to a desired temperature with an integrated Peltier element, while microscopy images of each droplet are recorded at fixed time intervals. The microscopy images are then manually processed to calculate the ratio of droplets that crystallize as a function of time and the effective nucleation rate at a prescribed supersaturation. The spatial and temporal temperature distributions across the microfluidic device are characterized to ensure accurate induction time measurements. The presented microfluidic platform ensures mixing of droplets with serpentine micromixers. The degree of mixing is controlled by the number of bends that droplets travel through in an experiment. We first characterize the nucleation kinetics as a function of supersaturation and droplet volume in static microfluidic experiments. Then, we elucidate the intertwined roles of droplet motion and mixing in microfluidic induction time measurements by comparing three different microfluidic induction time experiments referred to as “static”, “moving”, and “mixing” showing distinct behaviors. Moreover, we compare our microfluidic experiments with well-mixed, milliliter volume, turbidity-based measurements in the context of classic nucleation theory. We observed that the repeated microfluidic induction time measurements under identical conditions produced different cumulative nucleation probability curves particularly for experiments with moving droplets. We attribute this low reproducibility to details of the experimental procedure particularly the history of the droplets prior to temperature quench. We hope that the reported experimental procedures discussed in detail provide a learning experience for future studies. Our results highlight the relevance of mixing and droplet motion in microfluidic induction time measurements and open new alleys of investigation at the intersection of fundamental crystallization phenomena, wetting physics, and hydrodynamics.

## Materials and Methods

### Chemicals

All of the chemicals used in this study were purchased from suppliers and used without further modification. The chemical used and the suppliers are mineral oil (CAS: 8042-47-5, Sigma), KCl (CAS: 7447-40-7 CIK), and fluorescent dye rhodamine (CAS: 81-88-9, Sigma). All of the KCl solutions were prepared on a weight-by-weight basis by a dissolving a given amount of KCl in weighted deionized water. The deionized water was acquired from a Millipore device (ELGA PURELAB, resistivity: 18.2 MΩ·cm at 23.6 °C).

### Microfluidic Device Design and Manufacturing

**Figure 2:**
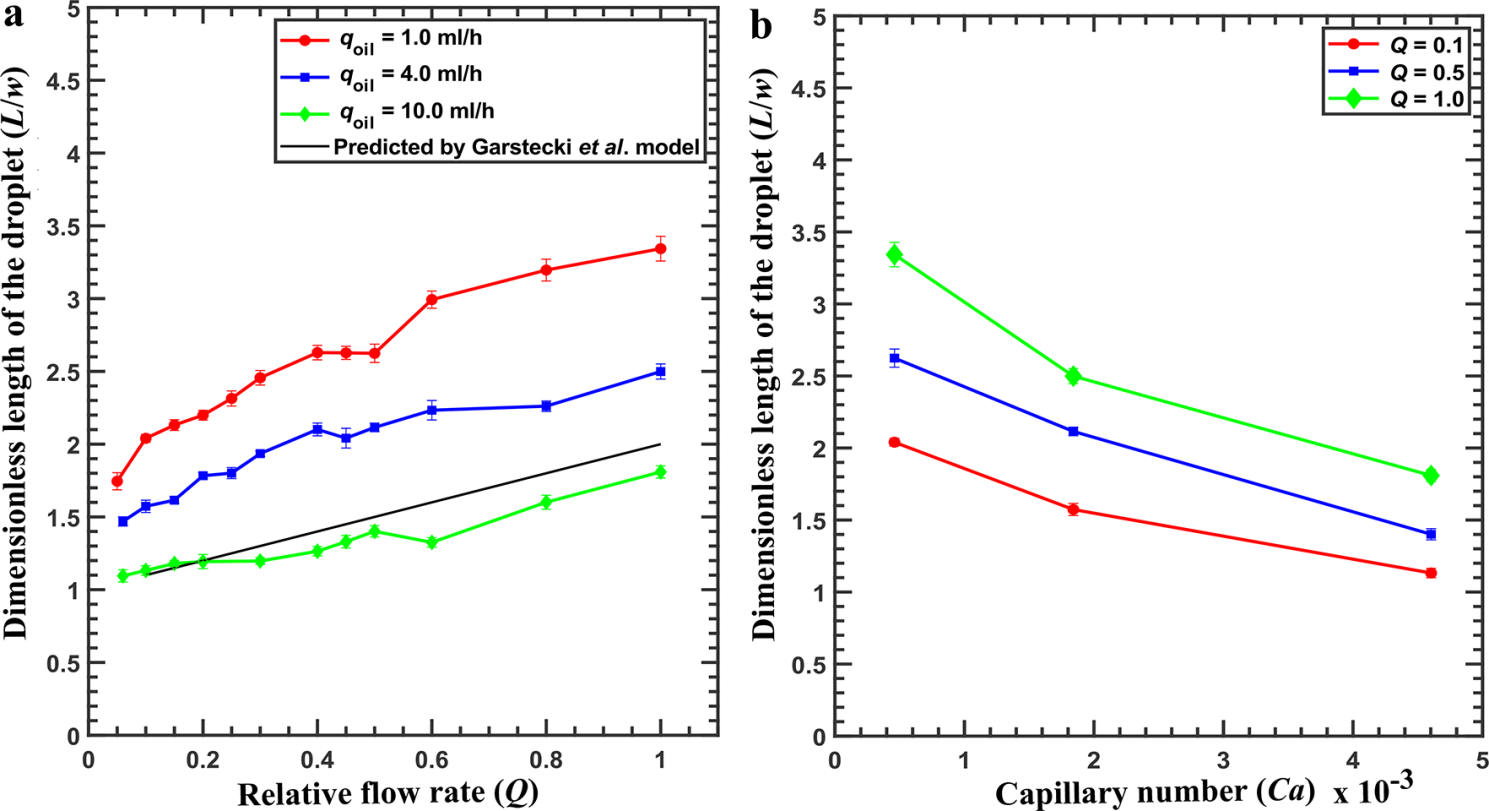
Controlling droplet size: the nondimensionalized droplet size, *L*/*w*, is presented as a function of relative flow rate, *Q*, in panel a and capillary number, *Ca*, in panel b at different flow rates, *q*_o_, of the continuous oil phase. Error bars represent the standard deviation of about 15 droplets for each case.

**Figure 3:**
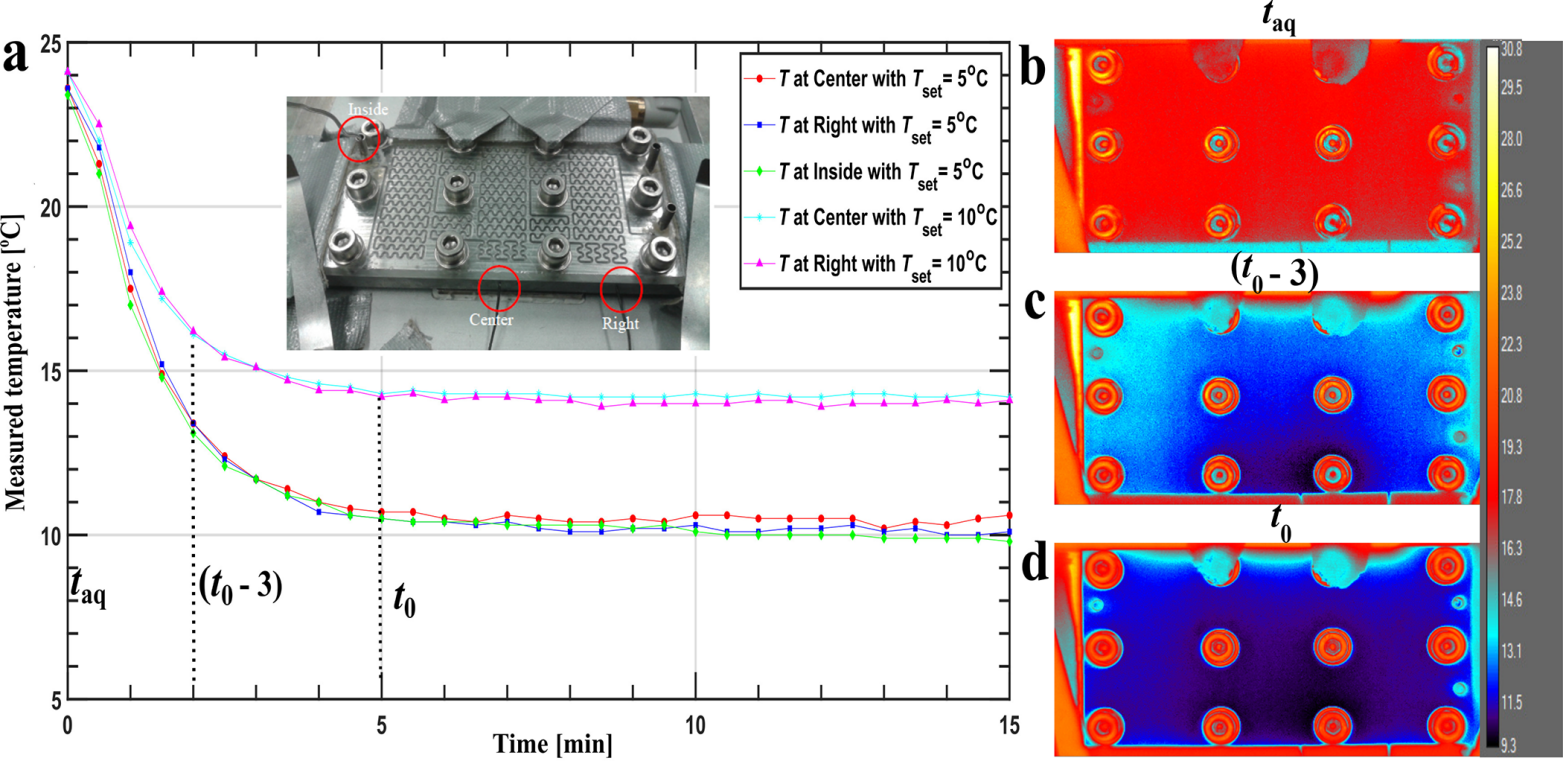
Characterization of spatial and temporal temperature distribution: (a) Temperature variation as a function of time measured with thermocouples throughout the experiment. Thermal equilibrium is achieved within 5 min. The inset shows the microfluidic chip resting on the Peltier cooling stage with three thermocouples placed (red circles) at three different positions on the Al plate. Panels b–d show the spatial temperature distribution probed by a thermal camera at different times after cooling *t*_aq_ = 0 (b), *t*_0_ – 3 = 2 min (c), and *t*_0_ = 5 min (d).

**Figure 4:**
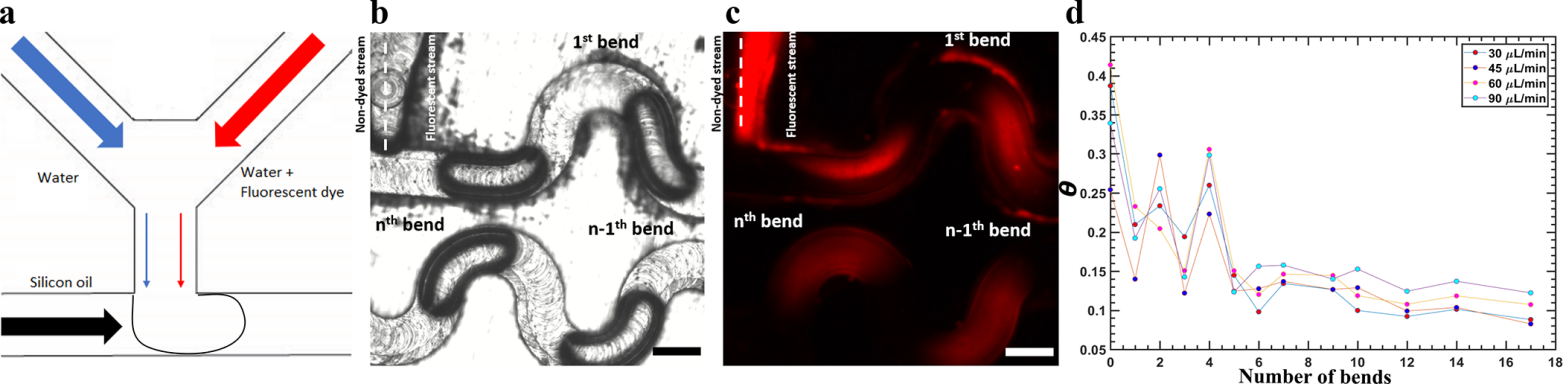
Quantification of droplet motion induced mixing: (a) Illustration of the microfluidic device designed specifically for mixing quantification experiments, (b) bright field microscopy image of droplets in serpentine micromixers, (c) fluorescent microscopy images of droplets showing the fluorescent dye homogeneously distributed as the droplets move through the micromixers, (d) Danckwerts number, θ, versus the number of bends a droplet moves through indicating complete mixing after six bends under canonical flow rates.

**Figure 5:**
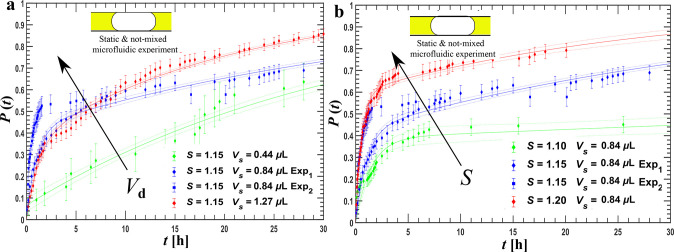
Induction time measurements for the “static and not mixed” microfluidic experiment. Panel a shows the cumulative probability distribution function, *P*(*t*), for different droplet volumes at fixed supersaturation, *S* = 1.15. Panel b shows *P*(*t*) for static microfluidic experiments with different supersaturations at a fixed droplet volume *V*_d_ = 0.84 μL. Error bars are those given by eq 1 in the Supporting Information.

**Figure 6:**
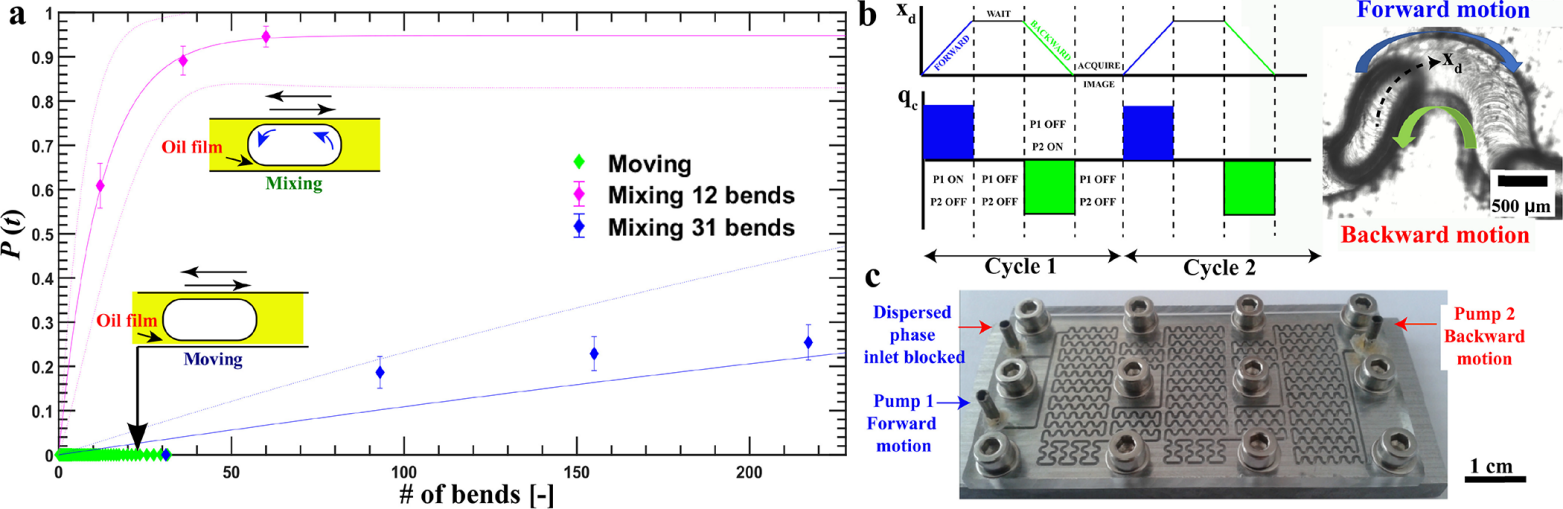
Influence of droplet motion and mixing on nucleation kinetics: Panel a shows cumulative distribution function, *P*(*t*) at fixed supersaturation, *S* = 1.13 for “moving”, mixing 12 bends”, and “mixing 31 bends” experiments. The error bars are standard deviation. Panel b shows the droplet movement. *x*_d_ is the displacement, and *q*_c_ is the continuous phase volumetric flow. The arrows and pumping sequence are coded: blue is forward and green backward motion, induced by alternating between two pumps. Panel c illustrates the pump connections to the microfluidic chip used for the droplet motion.

The proposed microfluidic tool consists of two sections: a droplet generation section with a T-junction and an observation and mixing section with serpentine passive micromixers as shown in (Figure 1a,b). The T-junction (Figure 1b) produces droplets of controlled size, and the serpentine micromixer ensures that the contents of the droplets are mixed as they move through the bends via baker’s transformations.^14,34^ The device is made from an aluminum plate with the channel grooved on it with micromachining. An acrylic glass plate is fixed on top with the help of aluminum screws, thus covering the channel. The device design ensures that (a) a maximum number of droplets are accommodated within a standard glass slide area of (25 × 75 mm), (b) the droplets are cooled down as rapidly and homogeneously as possible because of the high thermal conductivity of aluminum, (c) moving droplets are observed with video microscopy and mixed to the desired extent through asymmetric recirculation flows inside the droplets also known as baker’s transformations.^14,34^ The microfluidic devices used for most static experiments, which are shown in Figures 1–5, have a channel width of 0.5 mm and a channel depth of 1 mm as shown in Figure 1c. Each bend is 1.7 mm. The microfluidic device used for the mixing experiments, shown in Figure 6, is the same, except for a different channel depth of 0.5 mm. This changes the volume and shape of the droplets, as well as the interface with different surfaces. It should be noted that the device consists of two parts made of different materials, hence having distinct wetting properties and roughness. The aluminum plate is rougher than the acrylic glass plate. We hydrophobized both surfaces with a silanization agent according to the procedure described in Schuurmans et al.^36^ to ensure that the continuous phase wets all the confining surfaces uniformly.

### Characterizing the Droplet Formation

To explore our ability to control the droplet size in the our device, we measured the droplet size distribution with optical microscopy for various dispersed and continuous phase flow rates. For this study, mineral oil and deionized water are used as a continuous phase and dispersed phase, respectively. The experimental setup is represented in Figure 1a, which shows two microfluidic pumps to infuse continuous and dispersed phase liquids. At the T-junction, the dispersed liquid is fed perpendicularly with respect to the continuous phase liquid flow. During experiments, the relative flow rate of water to oil, *Q*, defined as *Q* = *q*_w_/*q*_o_ is varied between 0.5 and 1.5. The oil flow rate, *q*_o_, is kept constant at either 1, 4, or 10 mL/h, and the water flow rate, *q*_w_, is changed to reach a given *Q*.

### Characterizing the Temperature Distribution

To characterize the spatial and temporal temperature distributions across the microfluidic device, we utilize thermocouples and a thermal camera (FLIR A655sc). The inset of Figure 3a shows the various positions where the thermocouples are placed on the microfluidic device. The measurements shown in Figure 3a indicate that the target temperature of 10 °C is reached within 5 min. Figure 3b shows thermal camera images at the start of the experiment at *t* = 0 (Figure 3b1), *t* = 2 min (Figure 3b2), and *t* = 5 min (Figure 3b3). These thermal images also incorporate the thermocouple results and show that the target temperature is reached within 5 min. Moreover, they point out that the spatial temperature distribution is homogeneous.

### Cleaning Procedure for the Microfluidic Device for Induction Time Measurements

The aluminum and acrylic glass plates were sonicated while they were kept immersed in an ethanol bath for 20 min. After being washing with deionized water, both plates were dried using dry N_2_ gas. Additionally, the tubing carrying the dispersed and continuous phases was flushed by ethanol and then by deionized water and then dried using dry N_2_ gas.

### Milliliter Volume Induction Time Measurements

Induction time measurements with 1 mL volume KCl solutions at different supersaturations were performed using a commercial turbidity-based device, Crystal16. All induction time experiments are performed at 10.0 ± 0.5 °C. The temperature profile used is identical to microfluidic experiments to facilitate fair comparison. Note that the stirring rate was reduced from 700 rpm during the cooling phase to 200 rpm when the constant supersaturation was reached. The same stirring conditions were maintained thereafter during the turbidity measurements.

### Microfluidic Induction Time Measurements

Aluminum plates with microfluidic elements including the T-junction and serpentine channel were covered tightly with acrylic glass plates by using pairs of screws and washers as shown in Figure 1c. The cleaned microfluidic device was placed over the Peltier element and held firmly over it. The temperature of the Peltier element was maintained at 40 °C for 30 min prior to the droplet formation. A clear solution of KCl with a targeted supersaturation ratio is prepared based on the solubility of KCl at 10 °C, i.e., 31.2 g of KCl per 100 g of water.^37^ The supersaturated solution was not filtered.

The aqueous droplets of KCl dispersed in mineral oil were produced at the T-junction as shown in Figure 1b. The dispersed aqueous phase and continuous oil phases were pumped into the microfluidic device with syringe pumps (Figure 1a) at the prescribed oil and KCl solution volumetric flow rates as *q*_0_ = 10, 4, and 1 mL/h and *q*_aq-KCl_ = 1, 2, and 6 mL/h, respectively. The magnitudes and ratio of flow rates determine the droplet size at T-junction as shown in Figure 2. The transfer tubing was immersed in a hot water bath kept at 40 °C to avoid crystallization during solution transfer or prior to application of the temperature profile in Figure 1e. Also the syringe carrying the aqueous KCl solution was kept at 40 °C with a microfluidic heater presented in Figure 1a. Microfluidic heater is a heating jacket designed for syringe pumps. After the droplet formation, the inlets were covered with parafilm to prevent evaporation.

The microfluidic device, filled with droplets of the required droplet size and supersaturation, was mounted onto an inverted optical microscope (Nikon TE) in a reflective imaging mode with the acrylic plate facing the objective. Then, Peltier element cools the chip from 40 to 10 °C with the rate of 20 °C/min and maintains the target temperature during the experiments as illustrated in Figure 1f. The supersaturated droplets in microfluidic device are monitored as a function of time using the automated microscope stage. The field of view illustrated with the red square in Figure 1a is smaller than the whole chip surface. The field of view is automatically moved across the chip and composite images of the entire microfluidic device is acquired automatically. Typical composite images at the start of the experiment and a later time with nucleated crystals are given in Figure 1d. In the initial 2 h composite images were taken every 5 min. At longer times, intervals the acquisition intervals were increased to 30 min and 1 h depending on the supersaturation. As shown in 1e, image acquisition was initiated before reaching the target temperature denoted as *t*_aq_, whereas *t*_0_ is the time when the system reaches the target temperature. Starting video acquisition at *t*_aq_ allows omitting of crystals forming before reaching the desired supersaturation from induction time calculations.

Lastly, the acquired composite images (Figure 1d) are processed manually by counting the number of droplets with crystals in a given time frame. Crystals form nearly instantaneously, and only a single crystal per droplet has been seen. They are 0.2–0.3 mm in size. The number of crystal emerging as a function of time is then converted to a cumulative probability distribution function, *P*(*t*), expressed as,
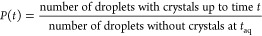
1We have specifically chosen the wording “total number of droplets without crystals at *t*_aq_” in eq 1 to emphasize the fact that droplets crystallizing prior to *t*_aq_ are not considered in calculating *P*(*t*).

### Models for Fitting Cumulative Distribution Functions

The cumulative probability distribution functions are fitted to two different models: (i) single exponential (eq 2) and (ii) two exponential (eq 3) using weighted least-squares curve fitting procedures implemented in Matlab software. The fitting equations are
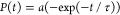
2
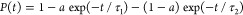
3where *a* is a prefactor, and τ is the induction time in eq 2. In eq 3, τ_1_ and τ_2_ represent two distinct nucleation time scales.

The parameter estimation procedure also delivers standard errors and confidence intervals in the estimates of the parameters, such as τ and *a*. These are the commonly obtained results from such procedures on the basis of a linearization of the model near the estimate. In some cases, approximate confidence intervals were obtained taking the nonlinearity into account. Typically, the time constants have errors on the order of 10% or more.

The conversion to the specific nucleation rates for these models are through the time constants, such that
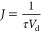
4where *V*_d_ is the mean droplet volume. Similarly, for the mixture of two-exponential distributions, *J*_1_ and *J*_2_ are obtained from τ_1_ and τ_2_. The error in *J* is due to the uncertainty in τ, on the order of 10% or more, and the volume. The droplet volume is approximated by the relation given in Musterd et al.^38^ for a given trapezoidal channel, whereby the droplet length is obtained from 10 observations in each case and has a variation on the order of 3%, which gives an insignificant standard error of the mean on the order of 1%.

### Experimental Quantification of Mixing

The mixing efficiency of serpentine passive mixers shown in Figure 1 is quantified according to methodology of Harshe et al.^35^ To this end, a new device with two dispersed phase inlets (as shown in Figure 4a) allowing mixing of two aqueous streams (one containing dye and one without) is designed. Except having the two inlets for the dispersed phase, the dimensions and geometry of this device are identical to the device in Figure 1. In the microfluidic device shown in Figure 4a,b, the first two aqueous streams (one carrying a fluorescent dye and the other free of it) are brought together as shown in microscopy images in Figure 4b,c. As the viscous forces dominate at microfluidic scale, the two streams can only mix through diffusion. Consequently, when they meet they do not mix immediately but stay as two laminar streams as shown in Figure 4c before they enter the serpentine mixer. Once the droplets enter the mixer, they gradually mix after each turn as shown in 4b,c, indicated by the gradual distribution of dye inside droplet.

We quantify the degree of mixing by quantifying the gradual change in fluorescent intensity distribution proportional to dye concentration as a droplet travels through bends with fluorescent microscopy. Before the droplet enters the bends, the fluorescent dye is concentrated in only on one side of the droplet as shown in Figure 4c. With each bend the droplets travel through, the fluorescent intensity appears to be become more homogeneous indicating mixing. The observed trend in Figure 4c, i.e., the fluorescent intensity distribution becoming more homogeneous with each bend, is quantified by the Danckwerts number, θ, here^35^ given by,
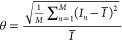
5

In eq 5, *M* is the number of pixels in area of interest, *I*_*n*_ is the intensity of *n*th pixel, and *I̅* is the average intensity in the area of interest. In essence, the Danckwerts number shows the deviation of light intensity from the average fluorescent dye intensity in the area of interest. The higher the Danckwerts number, the less mixed the droplet is. At a certain moment, the Danckwerts number is expected to become really small and constant, hence showing the droplet is completely mixed.

### Microfluidic Induction Time Experiments with Quantified Mixing

To isolate the effect of droplet motion and mixing, there are different microfluidic experiments performed at the same supersaturation, *S* = 1.13 and with the same droplet volume. The following experiments are preformed: (i) droplets are kept stagnant, (ii) droplets are moved less than a bend throughout the experiment, consequently not mixed, (iii) the droplets are moved over 12 bends, consequently well-mixed, and (iv) the droplets are moved over 31 bends. These are referred to respectively as “static”, “moving”, “mixing 12 bends”, and “mixing 31 bends”.

For all experiments numerated above, a potassium chloride solution with *S* = 1.13 at 10 °C was prepared. The potassium chloride was dissolved in water at 40 °C. It was kept at this temperature for at least 1 h to ascertain all potassium chloride dissolved. Before droplets are generated, mineral oil and the microfluidic device were both heated to 40 °C as well. The droplets are generated in the microfluidic device shown in Figure 1a as described in microfluidic induction time measurements. During droplet generation the KCl solution in the syringe and the microfluidic device were both kept at 40 °C with a syringe heater and a temperature controller. The microfluidic device is filled with droplets with an oil flow rate of 66.67 μL/min and an aqueous flow rate of 22.22 μL/min.

Once the droplets were formed and stored in the microfluidic chip at 40 °C, pump configuration, i.e., how the pumps are connected to microfluidic chip, was altered to move the droplets as shown in Figure 6c. The syringe pumps shown in Figure 1a were replaced with two syringe pumps carrying oil. One of the pumps is connected to the oil inlet and one other one to the outlet in Figure 1. The aqueous phase inlet is closed. With the program shown in Figure 6b, the two pumps, synchronized to pump in the same direction, move the droplets back and forward in the channel as shown in Figure 6c. The back and forward motion was done in a period of 80 s, which is needed for the pumps to build sufficient pressure and then move the droplets by covering a certain number of bends. The droplet motion was stopped for 100 s in every cycle (Figure 6b) to facilitate imaging of the droplets. In this total period of 180 s, the droplets are mostly stagnant and moving only a fraction of that period, which was roughly estimated. However, the number of bends was unambiguous, which is why the results of these experiments are expressed in bends as a measure for the actual mixing time. The actual travel time per bend was in the range from 0.5 to 3 s.

The microscopy images of the droplets were not taken at equal intervals, but the frequency of imaging was altered to efficiently use the limited hard disk space. The observations continued until the droplets started to coalesce or crystals moved out of the droplets. After a few back and forth movements, some droplets with grown crystals present started to block the channel causing coalescence. From this point, observations were halted, as the mixing was hindered, and the droplets were not unique single size entities anymore.

## Results

### Controlling the Droplet Size

The droplet size in microfluidic induction time measurements has been previously used to differentiate between homogeneous and heterogeneous nucleation in microfluidic induction time measurements.^3,17^ Consequently, the droplet size is a relevant parameter in microfluidic induction time measurements. We changed the droplet sizes by varying the flow rates of the dispersed water phase (*q*_w_), continuous oil phase (*q*_o_), and the relative flow rate (*Q* defined as *q*_w_/*q*_o_). Figure 2a shows the experimentally measured droplet size as a function of *Q* along with the theoretical prediction by Garstecki et al.^39^

Figure 2a illustrates how the droplet length, *L*, increases with *Q*, as the rate of droplet elongation prior to pinch-off is proportional to *q*_w_.^39^ Furthermore, the rate of droplet detachment is proportional to *q*_o_ leading to a shorter *L* at constant *Q*. This variation highlights the significance of flow rate magnitudes, as they influence the hydrodynamics of the droplet formation at the T-junction. At high oil flow rates, our results agree with theoretical predictions by Garstecki et al.^39^

In Figure 2b, the capillary number is defined as *Ca* = *μU*/σ, where μ, *U*, and σ denote continuous phase viscosity, characteristic speed, and surface tension, respectively. The characteristic velocity *U* = *Q*/(*wh*) with the cross-sectional area *wh* is channel width times height. As *Ca* increases, the normalized droplet size, *L*/*w*, decreases as the *Ca* number reflects the relative strength of viscous forces to surface tension. In other words, as the viscous shear forces increase, the droplets become smaller. The droplet formation in T-junctions is considered to be a balance between viscous, inertial, and surface tension forces.^40^ At low Reynolds (*Re* = *ρUw*/μ with ρ the continuous phase density) and *Ca* numbers, we expect *L*/*w* versus *Ca* at different *Q* values to collapse.^39,41^ However, our systems deviate from this behavior as shown in Figure 2b. Our experiments are conducted at *Ca* smaller than 5 × 10^–4^. However, because of the relatively large channel width, approximately 1 mm, *Re* is on the order of 50 in our experiments. We attribute the lack of collapse in Figure 2b to inertial effects.^14^

### Spatial and Temporal Temperature Measurements

The calibration of the temperature controlling system is essential as this work involves cooling crystallization. Induction time measurements are performed based on the KCl solubility data at 10 °C, for which the calibration is done. As shown in the inset in Figure 3, three thermocouples (their position is indicated with red circles) are used to measure the temperature on three different locations of the aluminum plate. From Figure 3a, we learned that to reach the target of 10 °C, the Peltier controller element has to be set to a lower temperature setting of approximately 5 °C. We attribute this to imperfect contact between the heating stage and the microfluidic chip. More importantly, both the thermocouple measurements in Figure 3a and IR camera measurements in Figure 3b–d indicate that the time required for the device to reach a spatially homogeneous temperature of 10 °C is approximately 5 min. These measurements allowed us to accurately predict the time required to obtain uniform and constant temperature that corresponds to *t*_0_ as shown in Figure 3d. To summarize, these spatial and temporal temperature measurements illustrate the temperature profile used in microfluidic induction time measurements as indicated in Figure 1e. It shows that the desired constant supersaturation is achieved 5 min after the start of acquisition. Moreover, this point marks the start of the induction time measurements.

### Quantification of Mixing

The internal mixing in the droplets is observed after each bend in the microchannel by monitoring the spatial fluorescent intensity distribution in droplets illustrated in Figure 4. These experiments are performed at different flow rates but with the same relative flow rate *Q* = 1/3. The mixing after each bend is quantified by the Danckwerts number, θ, as defined with eq 5.

Figure 4d shows the Danckwerts number values reach a plateau after five bends, indicating that the droplets are then well-mixed. The Dean number in this case is on the order of one, which is low and implies that little mixing takes place in one bend. The experiment then puts a lower limit of five, which is independent of the flow rate as previously observed in the literature.^35^ Five bends correspond to a distance of approximately 8.5 mm.

Fluorescent images in Figure 4b confirm this observation. We attribute the oscillations in Figure 4d to the fact that the droplets are three-dimensional objects going through baker’s transformation; yet, we observe only a projection of the droplet in our frame of view. The intensity measured can decrease, with dye flowing from the front of the droplet to the back of the droplet. Similar behavior is also seen with Harshe et al.^35^ but to a smaller extent.

### Static Microfluidic Induction Time Measurements

Six different sets of microfluidic measurements have been performed, which together give the five different profiles induction time profiles in Figure 5a,b. The channel size during these static measurements was 0.5 × 1 mm^2^.

To model the data, we fitted 10 different models to *P*(*t*), namely, single exponential, Weibull, and two exponential, which are generally applied, and Generalized Pareto, Gompertz, Gumbel, extreme value, log–logistic, log–normal, and the Pound–La Mer distribution, which are also quoted in the literature.^3,16,42^ In the Supporting Information is shown that most often the Pound–La Mer and the two exponential are among the best fitting ones. Both describe the nucleation process equally well and have two time constants. However, statistically, the two cannot be distinguished. The choice was made to present here the results for the two exponential, as it is the simplest to interpret as the presence of two separate phenomena with distinct rates *J*_1_ and *J*_2_. The fitted values are given in Table 1. The fast component, *J*_1_, only affects the first part of the curve and is also subject to uncertainties in the number of nuclei lost before the final temperature is reached. The slow component, *J*_2_, is relatively better estimated and is therefore more meaningful.

**Table 1:** Two-Exponential Fit for Figure 5a,b: Parameters with 95% Confidence Intervals and Effective Rates of Nucleation, *J*, with Combined Uncertainty Originating from Fitting Procedure and Droplet Volume for Different Volume Static Microfluidic Experiments

	τ_1_ (min)	τ_2_ (min)	*a*	*J*_1_ (m^**–**3^ s^–1^)	*J*_2_ (m^**–**3^ s^**–**1^)
*V*_d_**(**μL)	Supersaturation S1.15, Figure 5a				
0.437	5.5	1980	0.07 ± 0.03	70 × 10^5^ ± 180 × 10^5^	19.3 × 10^3^ ± 1.6 × 10^3^
0.842	27	2300	0.42 ± 0.03	7.4 × 10^5^ ± 1.8 × 10^5^	8.5 × 10^3^ ± 1.1 × 10^3^
1.270	73	1200	0.35 ± 0.03	1.8 × 10^5^ ± 0.3 × 10^5^	10.6 × 10^3^ ± 0.6 × 10^3^
*S* (−)	Supersaturation *V*_d_ 0.842, Figure 5b				
1.10	87	18000	0.39 ± 0.05	2.3 × 10^5^ ± 0.7 × 10^5^	1.1 × 10^3^ ± 1.0 × 10^3^
1.15	27	2300	0.42 ± 0.03	7.4 × 10^5^ ± 1.8 × 10^5^	8.5 × 10^3^ ± 1.1 × 10^3^
1.20	34	1600	0.60 ± 0.02	5.8 × 10^5^ ± 0.5 × 10^5^	12.1 × 10^3^ ± 2.1 × 10^3^

To characterize the microfluidic platform, we first report the static induction time measurements as a function of droplet volume. More than 100 droplets with a varying volume (0.44 ± 0.02, 0.84 ± 0.02, and 1.27 ± 0.04 μL) at supersaturation *S* = 1.15 are produced to assess the effect of droplet volume on induction time, as presented in Figure 5a and the first part of Table 1. The droplets with the largest volume show the highest induction probability.

The fitted parameters in Table 1 show that the nucleation rates, expressed per volume unit, are not constant as would be expected from homogeneous nucleation following classical nucleation theory (CNT). Both the fast, *J*_1_, component and the slow, *J*_2_, component tend to decrease by volume.

Next, we investigate the influence of supersaturation ratio, *S*, defined as ratio of salt concentration divided by equilibrium concentration at experimental temperature. As expected, due to the greater driving force, faster kinetics are observed when *S* is the highest, as evident in Figure 5b. To analyze the effect, at least 200 droplets of fixed volume at varying supersaturation levels (*S* = 1.1, 1.15, and 1.2) were prepared. The exact number of droplets for each experiment is given in the Supporting Information.

### Nonstatic Microfluidic and Turbidity Induction Time Experiments

With the setup in Figure 1, three induction time mixing experiments are conducted to elucidate the influence of droplet motion and mixing on the microfluidic induction time measurements. Channel size during these experiments was 0.5 × 0.5 mm^2^. A static experiment was conducted as a comparison with the device of the previous section (see Figure S.4 in the Supporting Information; τ_1_ = 12.7 (min), τ_2_ = 4700 (min), *a* = 0.15 ± 0.02). The mixing experiments are performed with identical supersaturation generation procedures, i.e., with identical temperature profiles at fixed supersaturation.

In the first experiment, the droplets are moved less than a bend length to induce droplet motion but to avoid mixing, referred as “moving”. In the second and third experiments, the droplets are moved over respectively 12 and 31 bends to mix the droplet contents rigorously referred as “mixing”. A more detailed description is given in the Materials and Methods.

The available parameters of the limited exponential fit for these moving and mixing experiments are listed in Table 2. They are expressed in number of bends that the droplets have traversed. The duration of one bend is in the range of 0.5–3 s. This could not be ascertained, but the range does imply that the typical time constant is in the range of 1 s to 1 min. Because many droplets did not reach the acquisition stage, the fits were based on a limited number of points causing large uncertainties.

**Table 2:** Summary of Estimated Nucleation Parameters for the Moving and Microfluidic Mixing Experiments^a^

experiment	τ (bends)	*a*
moving	no nucleation observed in 16 h	
mixing 12 bends	12.0 ± 9.1	0.95 ± 0.12
mixing 31 bends	>900	∼1

a *V*_d_ = 0.356 μL and *S* = 1.13.

The experiment denoted as “moving” in Figure 6a shows an unexpected result. After 16 h, none of the droplets nucleated. Our initial expectation was that this experiment would show kinetics similar to the static case. Yet, it clearly shows considerably slower kinetics.

**Figure 7:**
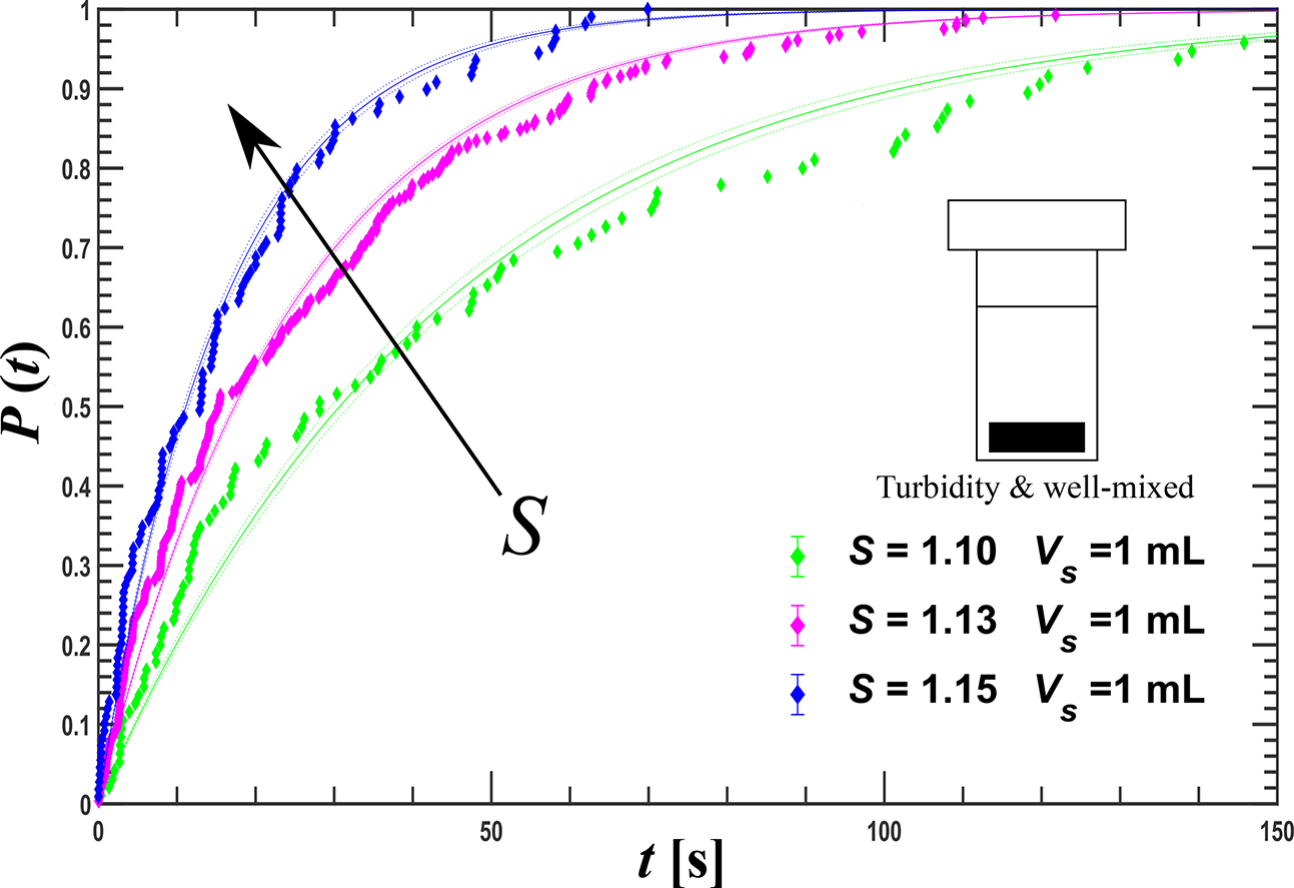
*P*(*t*) conducted at a well-mixed 1 mL volume at different supersaturations.

Additionally, induction time studies with a turbidity-based measurement in a well-mixed, one milliliter volume for a similar supersaturation range using Crystal16 are presented in Figure 7. When fitted with a two-exponential distribution, the mean induction time of the fast component proved to be on the order of 5 s, the sampling time of the observations. Besides, the cooling down period was on the order of 100 s, which implies that any process described by the fast component would have been completed already at the first measurement point. The fit results are listed in Table 3.

**Table 3:** Estimated Nucleation Parameters for a Two-Exponential Distribution Applied to the Turbidity Measurements (Figure 7)^a^

*S* (−)	τ_1_ (min)	τ_2_ (min)	*a*	*J*_1_ (m^**–**3^ s^**–**1^)	*J*_2_ (m^–3^ s^–1^)
1.10	0.20	1.03	0.235 ± 0.059	0.83 × 10^5^ ± 0.21 × 10^5^	16.3 × 10^3^ ± 1.0 × 10^3^
1.13	0.087	0.51	0.156 ± 0.015	1.92 × 10^5^ ± 0.21 × 10^5^	32.6 × 10^3^ ± 0.5 × 10^3^
1.15	0.028	0.32	0.134 ± 0.020	6.0 × 10^5^ ± 1.5 × 10^5^	52.5 × 10^3^ ± 2.5 × 10^3^

a Errors show 95% confidence intervals.

### Overview Nucleation Rates

**Figure 8:**
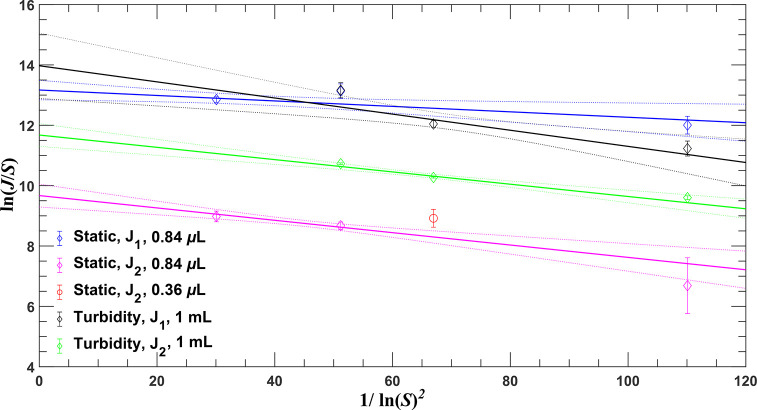
Test of classic nucleation theory: Plot of ln(*J*/*S*) as a function of 1/(ln *S*)^2^ for induction probability data on a 1 mL scale and the static microfluidic experiments.

According to the classical nucleation theory (CNT), the dependence of the nucleation rate *J* on the supersaturation is given by
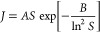
6where *A* and *B* denote the kinetic parameter and the thermodynamic parameter for nucleation^4,6^ respectively, which can be obtained from the straight line fit of ln(*J*/*S*) versus 1/ln^2^*S*. These are plotted in Figure 8 for both the fast and the slow component based on the numbers in Table 1. It is informative to see that effective nucleation rate values extracted from three different experimental procedures follow CNT. The estimated parameters *A* and *B* are presented in Table 4, which also confirms that the thermodynamic component, *B*, is positive such that the nucleation rate increases with supersaturation.

**Table 4:** Parameters *A* and *B* in eq 6 Applied to the Static and Turbidity Measurements

	*A*	*B*
microfluidic fast	66 × 10^4^ ± 21 × 10^4^	0.0090 ± 0.0073
microfluidic slow	2.0 × 10^4^ ± 0.8 × 10^4^	0.0205 ± 0.0081
turbidity fast	120 × 10^4^ ± 130 × 10^4^	0.027 ± 0.015
turbidity slow	11.8 × 10^4^ ± 4.4 × 10^4^	0.0204 ± 0.0057

Figure 8 also allows us to compare the nucleation rate from the two microfluidic devices used in this study. Consider only the more accurate slow nucleation rates, *J*_2_, at *S* = 1.13 (or 1/ln^2^(*S*) = 67 in the figure). The prediction for the 0.84 μL from the device with channel depth of 1 mm used for Table 1 is slightly less than the single measurement at 0.36 μL for the device with depth of 0.5 mm used in the mixing experiments (see Supporting Information for graph and data). The difference is significant but is explained by the factor of two difference in the volume, which was investigated in Figure 5a. So, the results of both microfluidic devices are comparable.

## Discussion

The first discussion point is the deviations of *P*(*t*) curves from single exponential behavior. Classical nucleation theory predicts that *P*(*t*) curves should be faithfully represented with a single exponential function. However, we find systematically different shapes of the probability curves which are best described by a sum of two exponential on the basis of their fit to the data; see Table S.1 in the Supporting Information. Such *P*(*t*) curves well-represented with a sum of two exponentials are considered to be a product of a slow component and a fast component. The slow component may be attributed to the primary nucleation process, while the fast component may be interpreted as another process causing the deviation from the single exponential.

For microfluidic *P*(*t*) curves, the fast component could be explained by nucleation of a different polymorph.^31^ As we do not individually characterize the form of each crystal contributing to *P*(*t*), this possibility can not be entirely ruled out. Another possibility is that the fast component originates from potent heterogeneous nucleation on the interfaces particularly channel walls. The third option has to do with the operation prior to *t* = 0. There, the droplets are moved into the channel with a Bretherton film surrounding it, and then it is cooled down, but this film takes time to squeeze out. So there will be a change in the nucleation rate due to this effect until the film has completely disappeared. The fourth option is that the volume of the drops havs a distribution. Dos Santos et al.^16^ has shown that the observed curve approximates a Generalized Pareto distribution, which can fit the data here as well (see Table S.1 in the Supporting Information). Finally, nonclassical nucleation could occur in the droplets in which nucleation occurs in two coupled steps. The formation of an unstructured dense phase followed by the nucleation of a structured crystal in the dense phase, however, was not observed in our experiments but might also require more sophisticated measurement techniques. In the case of the turbidity experiments, the initial fast components could be caused in our experiments by the sudden reduction in the stirring rate, so it has more to do with an operational switching effect.

Next, we discuss the effect of droplet volume on the effective nucleation rate. CNT, see eq 6, predicts that with the constant *S*, the homogeneous nucleation rate should remain the same irrespective of the system volume. Our result contradict this expectation based on the values presented in Table 1. The results of the experiments presented in Figure 5a show that the slow component of the nucleation rate *J*_2_ decreases with a larger droplet volume. A similar trend was reported by Steendam et al.^32^ who focused on the effect of scale up for well-stirred experiments with volumes ranging between 10 and 660 mL. Steendam et al.^32^ showed that as the volumes went down, the nucleation rates went up. We observed similar trends for static microfluidic experiments with three droplet volumes ranging between 0.44 and 1.27 μL. Steendam et al.^32^ suggested that a possible explanation could be difference in the shear rates as the volumes were changed. However, this explanation can not be used for our case as our measurements were carried out when the droplets were stationary. Moreover, the volumes in our experiments are significantly smaller. The large surface to volume ratio in our experiments might explain that the heterogeneous nucleation could play a more prevalent role. Moreover, this is in agreement with the work by Sear^3^ and Leisner et al.^43^ as well, who interpreted the dependence of the nucleation rate on the droplet volume as a sign of heterogeneous nucleation in the context of the classical nucleation theory. This interpretation is also in line with the hypothesis we offer for higher nucleation rates observed in static experiments given Table 1 compared to moving experiments in Table 2.

The next discussion point is the CNT analysis given in Figure 8. In all cases, the points involved are on a straight line within error bars. This applies to both the fast and the slow component in both the microfluidic and the turbidity case. The interesting observation is that the slope of the lines, represented by the thermodynamic parameter *B*, are all of the same order with a weighted average of 0.020 ± 0.003. This implies that all processes are possibly influenced by the same energy differences. Table 4 shows that there is no measurable difference in *B* between the microfluidic and turbidity observations. This confirms also the conclusion of Rossi et al.^29^ for adipic acid that flowing and internal mixing do not influence the thermodynamic parameter. Also the results of Nappo et al.,^30^ who compared the nucleation rates from their microfluidic device with those obtained in a turbulent vials of milliliter size, gave a comparable picture. The static microfluidic droplets give a lower rate, while the mixed droplets gave much higher nucleation rates than the turbidity-based experiments, despite the much lower estimated shear rates in the moving droplets in the microfluidic device.

Turbidity measurements have been done here as a reference for the microfluidic nucleation rates. The *J*_2_ of static microfluidic rates are a factor of seven lower than those from the turbidity observations over the whole range of supersaturations based on the fits presented in Figure 8. The decrease in volume would induce an increase in the nucleation rate (as in Table 1), while the absence of mixing/turbulence due to stationary droplets would induce a decrease in the nucleation rate as has been found by Rossi et al.^29^ The volume effect is apparently considerably larger than the droplet motion and hydrodynamic effect. The *J*_1_ cannot be usefully compared as the uncertainties are too high, and its cause could also be instrumental. The nucleation rates for mixing are one or two orders of magnitude higher than the turbidity observations (Table 2). Apparently, in this case, the hydrodynamic effect dominates, which might be explained by the intensive mixing that the droplets undergo in the serpentine channels during the well-mixed operation.

Another issue worth discussing in Figure 6 is the lower number data points and shorter experimental times for the mixing experiment compared to other two data points. This is because of the two complications developed during the experiments, droplet coalescence, and crystals left behind by moving droplets blocking the channel. The first complication of droplet coalescence emerged due to some droplets getting pinned on imperfections due to contact angle hysteresis.^44^ The pinned droplets then coalesced with moving droplets during day-long experiments. These imperfections may be either artifacts of machining of channels or stretches coming from the handling of microfluidic setup. The second complication emerges when the nucleated crystals sediment. The sedimented crystals are left behind as the droplets move across the bends. These crystals not only change the hydrodynamic resistance and hence the droplet velocities but also block the channels. Coalesced droplets or droplets which came in contact with crystals left behind were excluded from the microfluidic induction time results. Overall, the experiments involving droplet motion and mixing are harder to conduct than the static experiment. Because of coalescence, increased hydrodynamic resistance introduced by crystals formed in the channel, and higher uncertainties and shorter experimental times are recorded in Figure 6.

One may also consider what other microfluidic designs might be recommended to identify the influence of distinct mixing behaviors on *P*(*t*). An interesting design is a version of our design without serpentine channels. Comparing an experiment in a version of our design with only straight channels (no serpentine geometry) where the droplets are moved back and forth just like the mixing experiments would be an interesting future study. Such design will allow answering questions such as, Do the flow patterns of mixing influence the observed nucleation kinetics? As with straight channels, one would expect to have two symmetric circulating vortexes. Hence, the influence of mixing patterns might be deconvoluted.

The final discussion point is the interpretation of the experiments presented in Figure 6. As pointed out in the Results, significantly different *P*(*t*) curves are observed for “static”, “moving”, “mixing 12 bends”, and “mixing 31 bends” conditions. The expectation is that any induction time would shorten in this order. However, the “moving” case shows no nucleation, while the “static” case does, on the order of minutes or hours. The “mixing 31 bends” case has slower nucleation than the “mixing 12 bends” case. So far, the only hypothesis that could contribute to this is the history of the droplets prior to cooling down, as—with hindsight—this was not a fixed protocol nor recorded. Our results support the earlier found conclusion^45^ that moving droplets are surrounded by the Bretherton film. When the channels were filled, the droplets moved into place with this protecting film. Then, the droplets were kept stationary for some time during which the Bretherton film was squeezed out slowly. Subsequently, the mixing experiment starts, and the film builds up again. The result is that the observed initial induction time is the effect of mixing and the dynamics of varying Bretherton film. The latter is also determined by the prior protocol. From these experiments, we can so far only conclude that the hydrodynamics associated with mixing significantly reduces the induction time and that these experiments need a fixed protocol to avoid the dynamics of the Bretherton film.

Nappo et al.^30^ compared the nucleation kinetics for static and moving, nonmixed droplets of para-amino benzoic acid. They observed the enhancement of the nucleation rates by more than two orders of magnitude with the droplets in motion. They suggested that the shear rate might be responsible for this result. Moreover, they observed that, beyond a certain threshold limit, a higher shear rate adversely affected the kinetics. This could be an explanation for the results of our mixing experiments. For the mixing case, the shear rate would be in the optimum window, and hence the highest nucleation rate was obtained compared to turbidity-based measurements. However, for the moving case, the shear rate could be out of that optimum window and as a result nucleation might get hindered. Both Rossi et al.^29^ and Nappo et al.^30^ hypothesized that the probable cause of an enhanced nucleation rate could be the improved frequency of the collision among the mesoscale clusters. Particularly, Rossi et al.^29^ postulated that the attachment frequency was enhanced for the droplets in motion due to the internal recirculation taking place with the droplet motion. For recirculation, mixing needs to take place. We claim that movement in a microfluidic channel only causes a thin oil film in between the droplet and the channel, as described by Bretherton.^45^ This thin film literally lubricates the wall–droplet interface during movement. When the observations are made, the droplets are stagnant for some 100 s, but the breakup time is estimated to be much longer based on the work of Kreutzer et al.^46^ We employed a passive mixer so-called the serpentine geometry of the channel to ascertain whether actual mixing is taking place. Moreover, the mixing efficiency upon droplet motion through serpentine channels was confirmed with fluorescent measurements. This micromixing can improve mass transfer and therefore increase the time of observation of the crystal that has resulted in a significantly high nucleation rate. Another important point distinction between our study and other aforementioned studies^29,30^ is the chemical nature of solutes studied. The solute used in our study is KCl, an ionic salt which completely dissociates in water, whereas para-amino benzoic acid and adipic acid, organic compounds, are used in other studies. We expect that the nature of the interaction between the microfluidic channel walls will be influenced by this fact.

We also compare and contrast advantages and disadvantages of the proposed microfluidic device to previously reported experimental systems^29,30^ focused on measuring nucleation kinetics under different fluid dynamic conditions. Rossi et al.^29^ and Nappo et al.^30^ used T-junctions and millimeter-sized capillaries where the supersaturation is created by moving droplets from one temperature bath to another one at a lower temperature. Moreover, the nucleated crystals are moved to a third temperature bath to let crystals grow to reach an observable size, at a temperature very close to the saturation. This experimental setup is easy to clean due to large diameter capillaries and modules. Furthermore, the third temperature bath ensures that no further nucleation occurs, but crystals are grown to a given size. Compared to this setup, the presented setup is more difficult to clean, and once the microfluidic device is produced, it is not possible to alter the design. Yet, the presented setup has certain advantages. First of all, all the droplets are observed at all times. This ensures that droplets nucleating before reaching the desired supersaturation can be detected and taken out of construction of the *P*(*t*) curve. As the cooling system is integrated into the microscope, the desired temperature hence supersaturation is quickly achieved within 5 min, and sharp images of nucleated crystals are acquired as shown in Figure 1. Our design also enables complete rapid mixing of the droplets compared to axisymmetric circulation rolls created in droplets moving in straight tubes used by Rossi et al.^29^ In the setup used by Rossi et al.,^29^ liquid droplets move along the channel at a constant speed, and the fluid within them circulates, giving rise to counter-rotating vortexes with closed streamlines. Yet, the symmetry of these counter-rotating vortexes is maintained, and the fluid element circulating can only exchange across closed streamlines via diffusion. Moreover, that setup had the limitation of the pressure difference that can be applied due to the longer coil, which is not the case in our design. In summary, our device provides better images enabling analysis of crystal shape and more homogeneous mixing.

## Conclusions

We present a microfluidic platform that chaotically mixes droplet contents using serpentine-shaped channels and an experimental methodology to independently control droplet motion and mixing in microfluidic induction time measurements. The temperature stabilization and mixing in the proposed platform take place on the minute scale as deduced from dedicated experiments. This is negligible compared with the hour scale that the nucleation takes place in the proposed platform.

Cumulative nucleation probability curves extracted from microfluidic induction time measurements could be adequately fitted with a two-exponential model. The long-term component is mostly well estimated and signifies actual estimation for nucleation rates. Independent of the experiment, it is found that the thermodynamic component of the classical nucleation theory is the same, 0.020 ± 0.003. The experiment where the droplets move but the contents are not mixed did not show nucleation, which could be due to the suppression of nucleation by liquid–liquid film shielding droplets from contact with solid channel walls. In experiments with well-mixed moving droplets, faster nucleation kinetics is observed, emphasizing the role of mixing in microfluidic induction time measurements. The results were compared to turbidity experiments, which are done with near perfect mixing.

In future work, we will focus on the automation of crystal detection, along with the experimental extraction of size per droplet and better control to avoid droplet coalescence. Experiments should also include the mean nucleation time as a function of the fluid velocity during mixing in order to help deconvolute nucleation associated with kinetics, movement, and mixing. Hereby, the cause of the fast nucleation component should be investigated. With these experimental challenges resolved, more replicates of the experiments, particularly with moving droplets, will be most informative. Moreover, we will focus on designing experiments considering the history of droplets prior to the temperature stage. Finally, a study involving more solutes, and a larger range supersaturation, would be advisable.

## Supporting Information Available

Summary of different distribution models used to fit the induction time measurement data for static, mixing, and turbidity experiments presented in a table and figures (PDF)

## List of Symbols

Symbol: [unit] description
*A*: [m^–3^ s^–1^] kinetic parameter in CNT
*a*: [−] fraction of fast component in a two-exponential function
*B*: [−] thermodynamic parameter in CNT
*h*: [m] depth of microchannel
*I*: [−] intensity of a pixel
*J*: [m^–3^ s^–1^] nucleation rate
*L*: [m] droplet length
*M*: [−] number of pixels
*P*: [−] cumulative probability distribution
*Q*: [−] relative flow rate
*q*_c_: [m^3^/s] volumetric flow rate of continuous phase
*q*_o_: [m^3^/s] volumetric flow rate of oil
*q*_w_: [m^3^/s] volumetric flow rate of water
*S*: [−] supersaturation ratio
*T*: [K] temperature
*t*: [s] time
*t*_obs_: [s] time at which the droplets are observed
*t*_aq_: [s] the time at which the image acquisition was initiated
*t*_0_: [s] starting point for the induction time measurement
*U*: [m/s] characteristic speed
*V*_d_: [m^3^] droplet volume
*w*: [m] width of microchannel
*x*_d_: [m] total droplet displacement
θ: [−] Danckwerts number
μ: [Pa·s] fluid dynamic viscosity
ρ: [kg/m^3^] continuous phase density
σ: [N/m] surface tension
τ: [s] time constant in exponential functions
