# Supplementary material for: Microfluidic Platform with Serpentine Geometry Providing Chaotic Mixing in Induction Time Experiments
Source: Cryst Growth Des. 2022 Jun 9;22(7):4072–85. doi: 10.1021/acs.cgd.1c01436 (PMC9264360; doi:10.1021/acs.cgd.1c01436)
Supplement: Supplementary file 1 — cg1c01436_si_002.pdf [file cg1c01436_si_002.pdf]

A microfluidic platform with serpentine geometry providing chaotic mixing in induction time experiments

Sameer D. Shingte,[†,§] Olav Altenburg,[†,§] Peter J.T. Verheijen,[‡] Herman J.M. Kramer,[†] and Huseyin Burak Eral[*,†,¶,‖]

†Process & Energy Department,Delft University of Technology, Leeghwaterstraat 39, 2628 CA Delft, The Netherlands.

‡Biotechnology Department,Delft University of Technology, Delft, The Netherlands.

¶Van't Hoff Laboratory for Physical and Colloid Chemistry, Debye Institute, Utrecht University, Padualaan 8, 3584 CH Utrecht, The Netherlands.

§Contributed equally to this work

‖Corresponding author

E-mail: h.b.eral@tudelft.nl

Supporting Information Available

Different fitting models

The choice of distribution models is made on fitting all the data to a large selection of models and selecting the best fitting one. The publications of Dos Santos et al.[1], Mealey et al.[2] and Sear[3] were taken as the source of models. Dos Santos et al.[1] used a distribution which in statistics is known as the Generalized Pareto distribution. The criterion used here is the root mean squared error, RMSE, or estimated standard error was used. The approximate experimental error, ϵ_k, here is based on the binomial distribution with a lower bound of one unit. This then leads to the weight, W_k, for data point k:

$$\epsilon_k = \max(\sqrt{P_k(1-P_k)/N}, 1/N) \qquad \text{and} \qquad W_k = 1/\epsilon_k^2, \tag{1}$$

where P_k is the cumulative distribution value and N the number of droplets without crystals at t_{aq}. When the used weight are based on a correct measurement error (here eq 1), the RMSE should be on the order of one. This is confirmed for the better fitting models in Table 1. The two exponential and Pound-La Mer, both involving two time constants, are most often among the best fitting models per measurement series. The ease of interpretation favored to choose the two exponential.

Figures 1, 2 and 3 show some examples of the fit obtained with the measurement series from Figure 5a and b and three models, simple exponential, two exponential and the Weibull model.

S.1: Summary of all experiments and distribution models. 'Total number' is the initial number of drops before saturation. 'Number' without nucleation at time t_0 where constant super saturation begins. At the end of observation some drops still have no nucleation observed. Given is the root mean squared error for each fit.

	Microfluidic static					Microfluidic mixed			Turbidity			Separate exp	
Figure	5a			5b		6a		S4	7			5a 0.84μL	
Supersaturation	1.15	1.15	1.15	1.10	1.20	1.13	1.13	1.13	1.10	1.13	1.15	1.15	1.15
Volume (μL)	0.44	0.84	1.50	0.84	0.84	0.36	0.36	0.36	10^3	10^3	10^3	0.84	0.84
Total number	80	352	245	206	170	123	92	118	207	497	635	212	140
Number	33	325	218	173	159	123	92	118	96	288	120	190	135
Number no nucleation	3	73	6	87	33	91	5	88	0	0	0	16	57
Mixing number bends							12	31					
One exponential	0.71	6.20	3.27	5.75	4.62	2.87	1.40	2.96	1.06	1.33	1.17	4.96	4.89
Stretched exponential	0.60	1.97	0.66	1.09	1.37	0.39	0.50	2.96	0.62	0.59	0.60	1.18	2.36
Generalized Pareto	0.66	1.93	1.42	1.01	0.52	0.34	0.25	3.62	0.69	0.86	0.92	1.50	1.43
Gompertz	0.67	2.87	1.97	1.45	1.01	0.92	0.08	2.98	0.75	0.92	0.97	2.48	0.59
Gumbel	1.38	3.57	3.87	2.66	4.00	1.51	1.75	3.73	3.09	5.33	3.15	3.03	5.55
Extreme value	0.52	2.75	1.15	2.27	2.21	1.19	na	2.20	1.04	1.65	1.25	1.81	3.46
Log-logistic	0.77	1.94	1.31	1.00	0.92	0.37	0.03	2.48	1.02	1.76	1.46	1.28	2.06
Log-normal	0.51	1.85	0.94	1.00	0.82	0.28	na	0.01	0.79	1.20	0.99	1.25	1.59
Two exponential	0.50	1.64	0.82	1.43	0.69	0.59	na	5.13	0.50	0.46	0.48	0.95	0.58
Pound-La Mer	0.54	1.64	0.63	1.43	0.69	0.58	na	3.63	0.63	0.85	0.56	0.98	0.58
Limited exponential	0.69	2.97	2.40	1.51	1.26	0.94	0.39	2.96	0.95	1.23	1.11	2.72	0.62

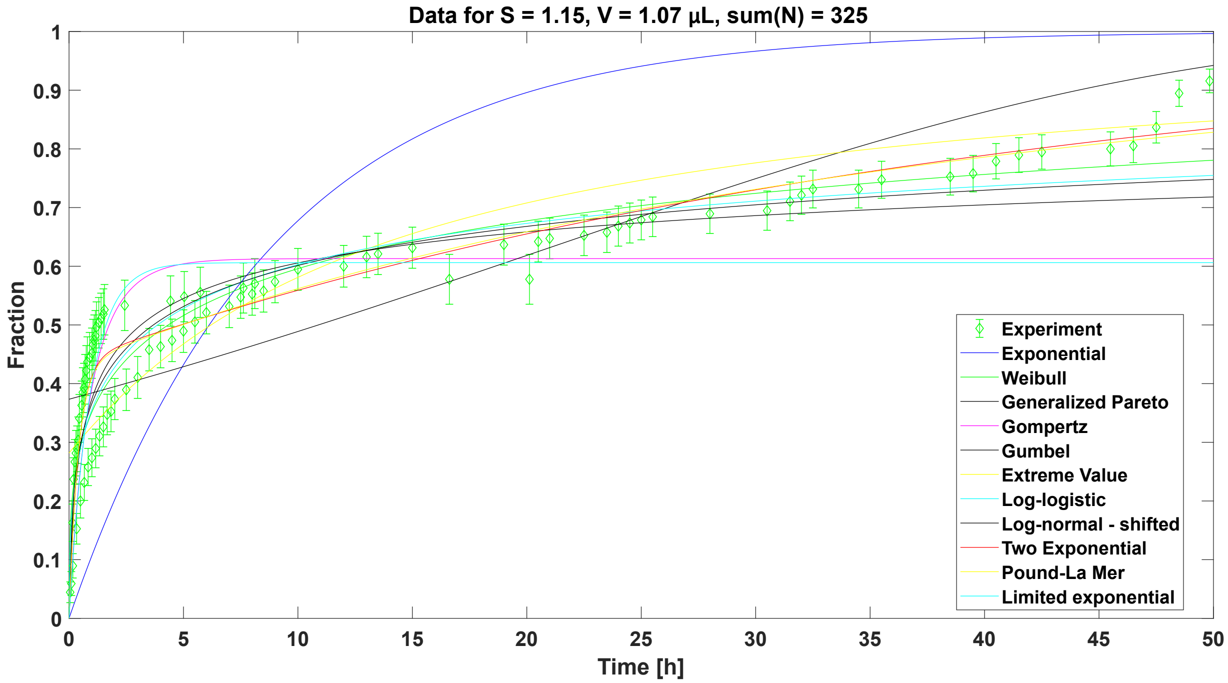

S.1: Different models fitted to an example of the static microfluidic data

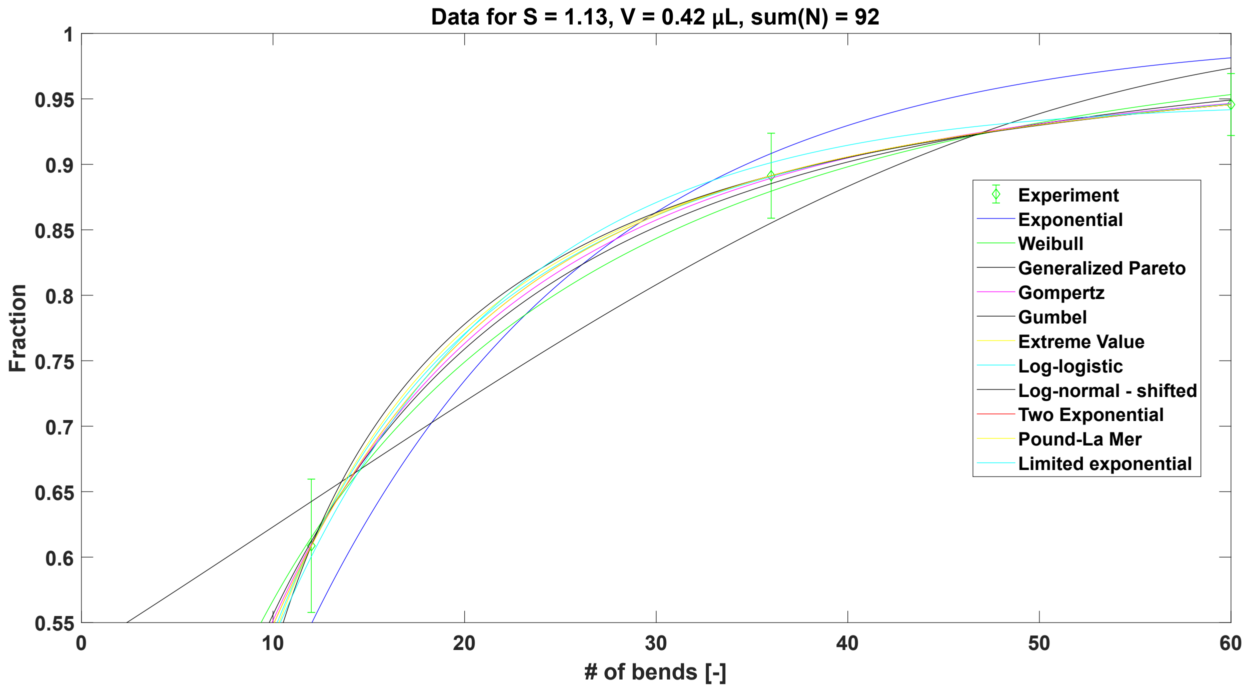

S.2: Different models fitted to an example of the mixing microfluidic data

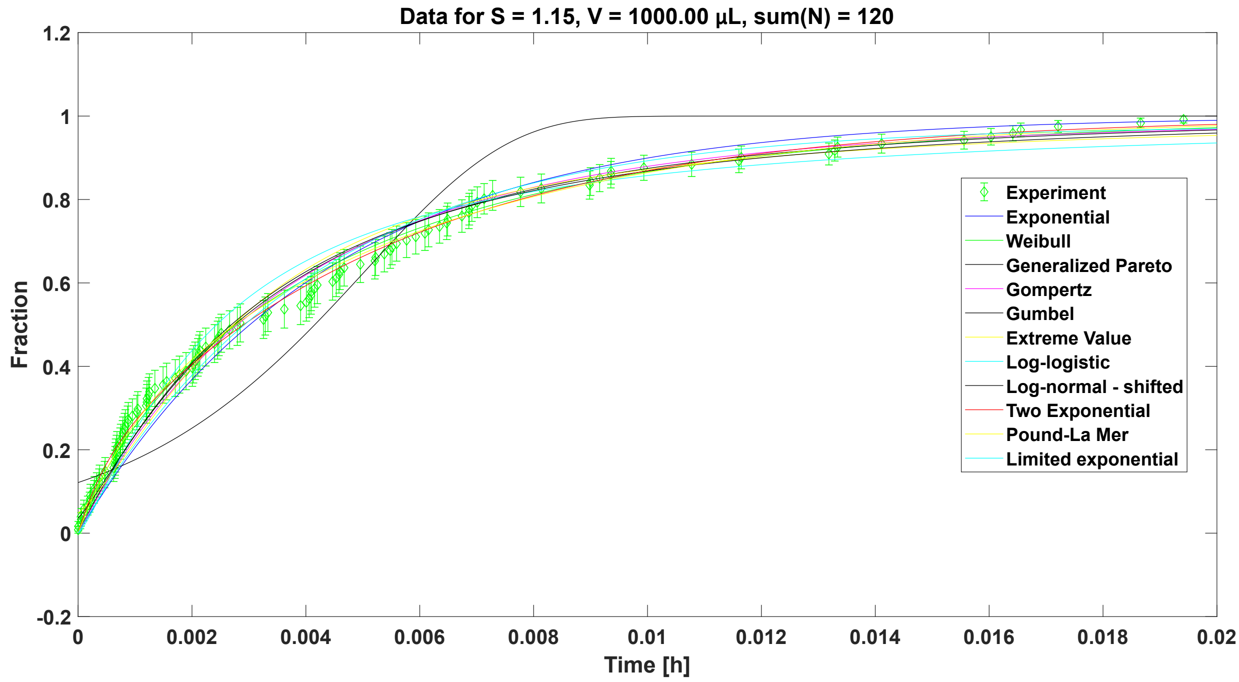

S.3: Different models fitted to an example of the turbidity data

Additional static measurement

The microfluidic device that was used for the mixing experiments, was also applied to do a single static experiment as a control or reference point. The measurement is given in figure 4. The fitted two exponential model gives $\tau_1 = 12.7$ (min), $\tau_2 = 4700$ (min), $a = 0.15 \pm 0.02$, $J_1 = 31 \times 10^5 \pm 11 \times 10^5$ and $J_2 = 8.4 \times 10^3 \pm 2.5 \times 10^3$.

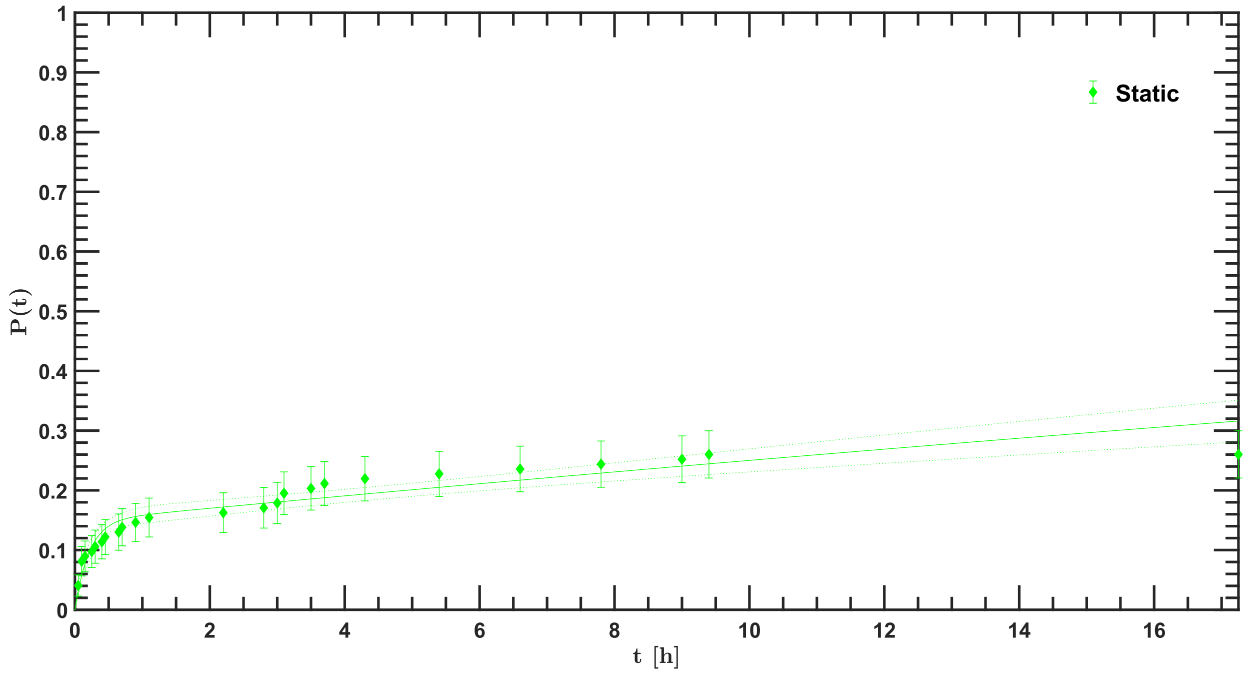

S.4: Static experiment on the device with the mixing experiments.

References

(1) Dos Santos, E. C.; Maggioni, G. M.; Mazzotti, M. Statistical Analysis and Nucleation Parameter Estimation from Nucleation Experiments in Flowing Microdroplets. *Crystal Growth & Design* **2019**, *19*, 6159–6174.

(2) Mealey, D.; Croker, D. M.; Rasmuson, Å. C. Crystal nucleation of salicylic acid in organic solvents. *CrystEngComm* **2015**, *17*, 3961–3973.

(3) Sear, R. P. Quantitative studies of crystal nucleation at constant supersaturation: experimental data and models. *CrystEngComm* **2014**, *16*, 6506–6522.
